# Supplementary material for: Human Heart Explant-Derived Extracellular Vesicles: Characterization and Effects on the In Vitro Recellularization of Decellularized Heart Valves
Source: Int J Mol Sci. 2019 Mar 14;20(6):1279. doi: 10.3390/ijms20061279 (PMC6471048; doi:10.3390/ijms20061279)
Supplement: Supplementary file 1 [file ijms-20-01279-s001.zip › ijms-450832 supplementary figures.pdf]

Supplementary Materials

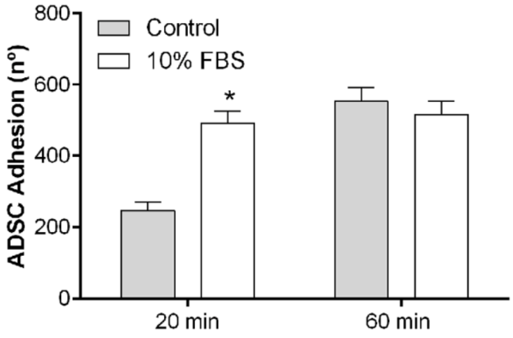

Figure S1. Standardization of the cell adhesion assay performed with ADSC cells. * $p < 0.05$.

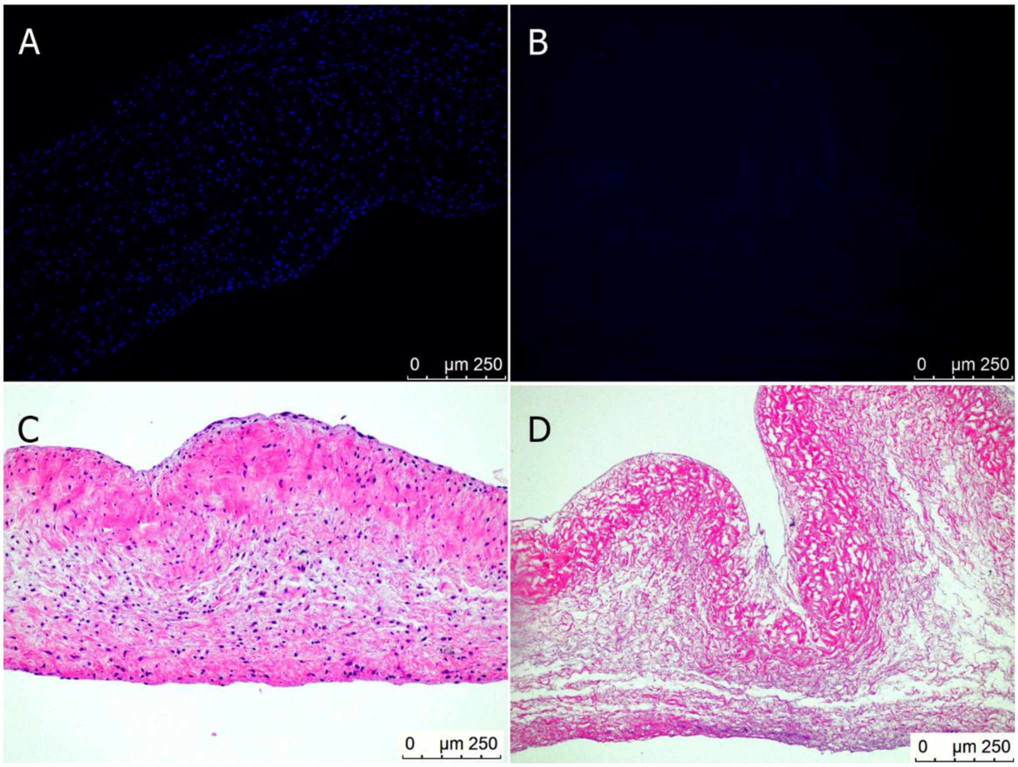

Figure S2. Analysis of the efficiency of decellularization. Representative images from native leaflets stained with DAPI (**A**) and H&E (**C**) and decellularized leaflets stained with DAPI (**B**) and H&E (**D**).

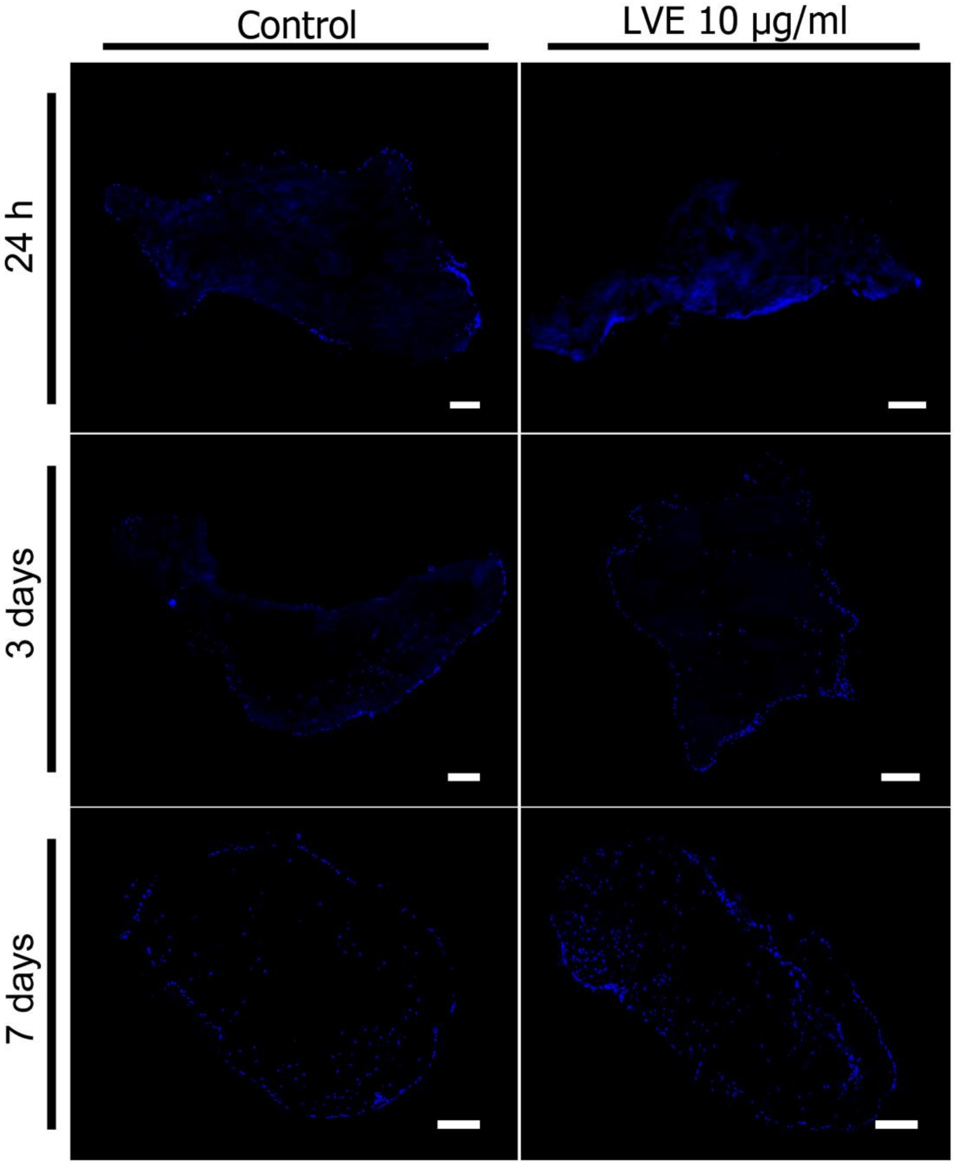

Figure S3. Representative images of recellularized fragments previously coated with 10 µg/ml LVE-EVs and cultivated with ADSCs for 24h; and recellularized fragments cultivated with ADSCs for 24h, stimulated with 10 µg/ml LVE-EVs and then cultivated for 3 and 7 days. Scale bar = 250 µm.

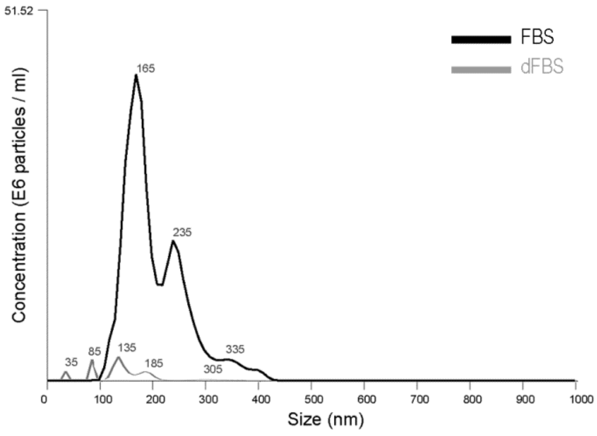

Figure S4. Representative graphic from NTA images of FBS and dFBS. The dFBS was obtained from the supernatant of FBS ultracentrifuged at 100,000 g for 20 hours. The samples were prepared at 10% (same concentration used in explant cultures) and diluted 20-fold in PBS before the analysis.
